# Medical tongue piercing – development and evaluation of a surgical protocol and the perception of procedural discomfort of the participants

**DOI:** 10.1186/1743-0003-11-44

**Published:** 2014-03-31

**Authors:** Bo Bentsen, Michael Gaihede, Romulus Lontis, Lotte NS Andreasen Struijk

**Affiliations:** 1 Center for Sensory Motor Interaction, Department of Health Science and Technology, Faculty of Medicine, Aalborg University, DK-9220 Aalborg, Denmark; 2 Department of Otolaryngology, Head & Neck Surgery, Aalborg University Hospital, DK-9000 Aalborg, Denmark

**Keywords:** Tongue control, Assistive device, Piercing, Surgical tongue piercing technique, Procedural pain, Expected pain, Perceived pain, VAS

## Abstract

**Background:**

A system providing disabled persons with control of various assistive devices with the tongue has been developed at Aalborg University in Denmark. The system requires an activation unit attached to the tongue with a small piercing. The aim of this study was to establish and evaluate a safe and tolerable procedure for medical tongue piercing and to evaluate the expected and perceived procedural discomfort.

**Methods:**

Four tetraplegic subjects volunteered for the study. A surgical protocol for a safe insertion of a tongue barbell piercing was presented using sterilized instruments and piercing parts. Moreover, post-procedural observations of participant complications such as bleeding, edema, and infection were recorded. Finally, procedural discomforts were monitored by VAS scores of pain, changes in taste and speech as well as problems related to hitting the teeth.

**Results:**

The piercings were all successfully inserted in less than 5 min and the pain level was moderate compared with oral injections. No bleeding, infection, embedding of the piercing, or tooth/gingival injuries were encountered; a moderate edema was found in one case without affecting the speech. In two cases the piercing rod later had to be replaced by a shorter rod, because participants complained that the rod hit their teeth. The replacements prevented further problems. Moreover, loosening of balls was encountered, which could be prevented with the addition of dental glue. No cases of swallowing or aspiration of the piercing parts were recorded.

**Conclusions:**

The procedure proved simple, fast, and safe for insertion of tongue piercings for tetraplegic subjects in a clinical setting. The procedure represented several precautions in order to avoid risks in these susceptible participants with possible co-morbidity. No serious complications were encountered, and the procedure was found tolerable to the participants. The procedure may be used in future studies with tongue piercings being a prerequisite for similar systems, and this may include insertion in an out-patient setting.

## Background

Current assistive devices for tetraplegics offer text input and control of a pointing device at different levels depending on the principles employed for their activation [1]. Further, they include different trade-offs related to user preferences such as unconstrained movements and aesthetic factors. Sip-n-puff systems provide a good proportional control (i.e., both speed and direction in real time) of a pointing device. This also applies to a chin joystick as well as a head control system. However, an onscreen keyboard is required to input text. Furthermore, face muscles have been used in systems generating on-off switch commands. However, users of these systems often report induced muscle fatigue and pain as main drawbacks. Speech recognition systems provide a remarkable text input and assure a minimal constraint for the user [2,3], but correction of false commands still needs to be addressed in the further development of these systems. Moreover, eye control systems provide a good proportional control of pointing devices, but limitations occur in low or changing light [4,5]. Brain computer interfaces have received increasing interest for control of assistive devices [6], though technical challenges still remain regarding the practical implementation in daily life due to a rather low detection rate of user intentions. Practically invisible when used, intra-oral tongue controlled systems have been developed using switch arrays, pressure, resistive, capacitive, magnetic or optical sensors embedded in a palatal brace [7-11]. Nevertheless, the extraordinary flexibility of the tongue has not been fully exploited for providing both direct text input and proportional control of a pointing device.

**Figure 1:**
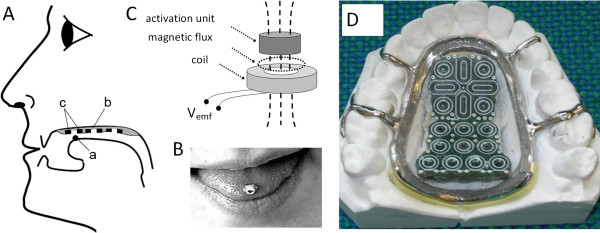
**Inductive tongue control system (modified from [**[12]**] with permission, © 2006 IEEE): (A) Placement of sensors (c), dental brace (b) and activation unit (a), (B) Activation unit attached to the tongue as the upper ball of a piercing, (C) Principle of activation for inductive sensors; perturbation of the magnetic field of the sensor by the activation unit induces an activation signal back into the sensor, and (D) The upper jaw dental brace placed on a plaster model.**

A new tongue control system has been developed at Aalborg University in Denmark. This assistive device gives individuals with severe sensory-motor impairment and lost function of the limbs a possibility to directly type text or to proportionally control a pointing device in order to control, e.g., electrical wheelchairs or personal computers with the tip of the tongue [12-19]. This system consists of a dental brace in the upper jaw encapsulating two pads of inductive sensors, a rechargeable battery and electronics. The inductive sensors are activated by changing their inductance using an activation unit consisting of a small cylindrical piece of soft ferromagnetic metal. This activation unit is attached to the tongue as the upper ball of a piercing (Figure 1), and it activates a given sensor in the sensor pad whenever it is positioned by the tongue at a specific sensor.

The induced activation signals are further processed and interpreted by the embedded electronics and sent wirelessly to an external unit to control the respective disability aids. A combination of induced signals from adjacent sensors makes it possible to continuously detect the position of the activation unit when gliding along the pad surface. Extended functionality has been obtained with both sensor pads when typing text (e.g., implementation of backspace, enter or arrows, besides a full alphabet keyboard similar to that of a mobile phone) in the so-called “text mode”, or when controlling the pointing device (e.g., right and left click and scrolling, besides a multidirectional and variable speed joystick) in the so-called “mouse mode”. A visual feedback continuously assists the user by showing the position of the activation unit when using the system which greatly improves the sensor activation and reduces the false activation rate.

The text input results of our system at rates between 1.68 and 2.94 correct words per minute (cwpm) have been obtained by subjects with tetraplegia after just three days of training [15]. These results may be much improved with longer training periods providing a better knowledge of the system and thereby improved user reaction time. Healthy subjects already accustomed to a cosmetic piercing of the tongue have been able to control the system from the first day in a surprising manner. Furthermore, in a study using a previous version of the system, induced cortical plasticity has been shown after a short period of training suggesting that the ability to perform specific tongue movements may be improved [16]. Alternative interfaces allow text input at rates of 12.1 cwpm for head control systems, 9.36 for eye control, 8 for mouse stick and 4 for tongue keypad systems. A speech recognition technique promises up to 120 wpm and a brain computer interface 12 cwpm [1]. Thus, the overall functionality of our tongue control system has shown promising results.

Furthermore, subjects have evaluated the tongue control system as easy to use and wear, and cosmetically acceptable. The subjects scored the system between 1 and 3 on a scale from 1 to 10 (1 = no discomfort and 10 = highest discomfort) for typing and pointing tasks as well as when talking or drinking with the mouthpiece [15].

The tongue piercing plays a vital role as the activation unit of the tongue control system. The word piercing is commonly used in connection with cosmetic body piercings involving the piercing of the human skin or mucosa and preparation of a duct in the underlying tissue with a sharp instrument followed by the insertion of a metal or composite ring or stud. Oral piercings mainly consist of two types: 1) a barbell consisting of a rod with a removable ball in each end or, 2) a labret consisting of a rod with a fixed disc at one end and a removable ball at the other end. The procedure of a cosmetic piercing insertion is usually not associated with medical procedures, and piercings are most often performed under unregulated circumstances in, e.g., tattoo shops and private homes, and there have been several reports about side effects like bleeding, swelling, infection, tooth fracture and abrasion [20-22]. The literature is sparse on information about piercing equipment, procedures and the magnitude of the discomfort of the procedure as well as during the healing period.

Since subjects eligible for tongue control systems are mostly tetraplegics, who are susceptible individuals with comorbidity such as decreased respiratory capacity and airway reflexes, the tongue piercing may pose an additional risk, thus demanding a safe and tolerable procedure. This paper describes a clinical technique developed for the insertion of a titanium barbell into the tongue including its safety precautions and complications. In addition, the discomfort perceived by the subjects during the procedure and the healing period was also evaluated.

## Methods and materials

### Volunteers

The study was aimed at subjects suffering from tetraplegia with various clinical backgrounds such as muscle dystrophy, cerebral palsy or spinal cord injury. The subjects were to have a good control of their tongue as well as normal cognitive skills and a high motivation for the study. Exclusion criteria were pregnancy, heart disease or other medical problems assessed to contraindicate the surgical procedure as well as a subsequent period of tests of the tongue control system. Patients with cognitive impairments as well as dental problems that could interfere with the study were also excluded.

Four tetraplegic subjects volunteered for this study. Two of these subjects suffered from previous traumatic injury of the spinal cord, one subject suffered from medullar compression due to a benign medullar glioma, and one subject suffered from childhood meningitis affecting the medulla. All four subjects were paralyzed from the neck, but exerted full normal control of the tongue with unaltered speech and normal cognitive skills. All subjects were able to use alternative assistant devices. The participation of the volunteers included the piercing procedure as well as the course of subsequent experiments with regard to learning its usage in the tongue control system. Information about participation was given both in writing and by oral explanation, and informed written consent including the publication of the individual study results was obtained from all subjects. The study was approved by The North Denmark Region Committee on Health Research Ethics (N2009-0013).

The overall study period comprised the initial period related to the insertion procedure of the piercing including a four week healing period and subsequently a four month experimental period where a series of performance tests were completed. Data related to these tests have been presented elsewhere [15]. Prior to the study period an oral examination was performed to obtain the dental status of each participant. Similarly, a second oral examination was performed at the end of the study period.

### The piercing and the surgical kit

**Figure 2:**
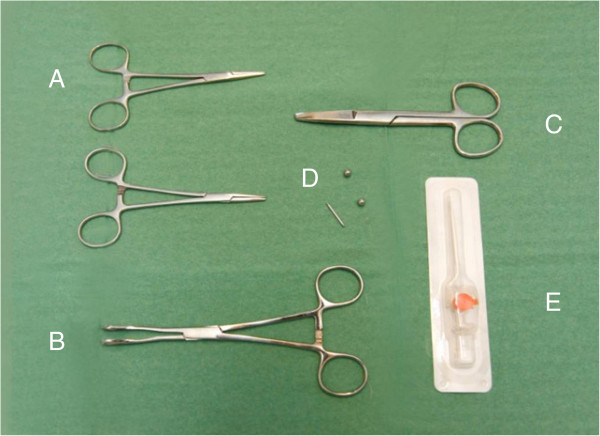
The surgical kit consisted of two needle holders (A), a tongue holder (B), scissors (C), piercing rod and two balls (D), and the BD Venflon™ Pro IV Canula 14 Gauge (= 2.0 mm) (E).

The current study chose a barbell piercing consisting of two metal balls and a rod (“nuts” and “bolt”). The parts were made from a medical grade Titanium alloy containing 6% Aluminum and 4% Vanadium (Ti6Al4V), which is a common and biocompatible alloy for dental implants [23]. The rod had a diameter of 1.6 mm, the lengths varied between 16 and 22 mm, and the diameter of the balls was 6.0 mm (Figure 2 items D). The piercing rods and balls were delivered from Star Piercing Company in Sweden together with documentation of the metal composition.

A surgical instrument kit was composed including two needle holders to fixate the piercing rod and fasten the metal balls. The holders were slightly modified forming a small bowl-shaped depression in their branches for an enhanced grip of the round surface of the balls (Figure 2 items A). Further, the kit included a set of tongue holding forceps (Foerster Ballinger forceps) as a mean of fixating the tongue. In order to reduce the pressure on the tongue, its branches were slightly adjusted making a 5 mm free space available when the forceps were closed (Figure 2 item B). Moreover, a pair of scissors was included (Figure 2 item C). All parts were cleaned in an ultrasonic bath, packed in sterilization pouches, and autoclaved in a dental vacuum autoclave. Finally, a sterile single use BD Venflon™ Pro IV Canula 14 Gauge (= 2.0 mm) was used to pierce the tongue (Becton Dickinson Infusion Therapy AB, Sweden) (Figure 2 item E).

### The surgical procedure

The participants were admitted for the insertion of the piercing and for 24 hours of observation at the Department of Otolaryngology, Head & Neck Surgery, Aalborg University Hospital, to ensure that the procedure was performed under professional conditions by trained health professionals (BB and MG) and to monitor any early adverse effects or complications.

The participants were introduced to the surgical procedure prior to the actual insertion of the piercing, and local anesthesia of the tongue was offered by means of a bilateral blockage of the n. lingualis by injection of Xylocaine 2%.

**Figure 3:**
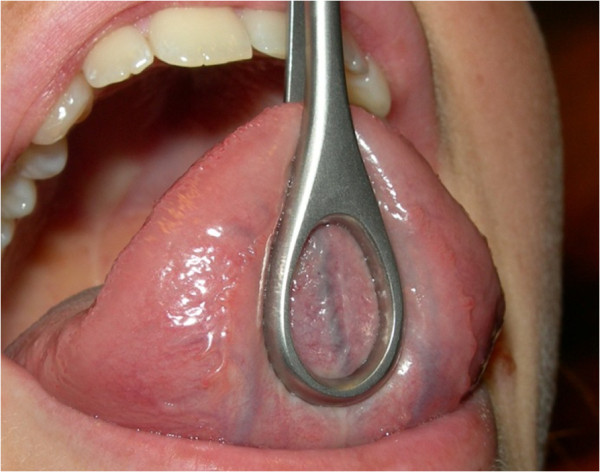
Relatively large blood vessel (vein) in the midline of the tongue (written consent for publication of this photo was obtained from the participant).

In order to create the best possible function of the activation unit, the entry point of piercing of the tongue was placed as near to the tip of the tongue as possible. However, an insertion too close to the tip would enhance the risk of drifting of the rod towards the periphery of the tongue and ultimately rejection of the piercing. The compromise was an insertion at around 20 mm from the tip of the tongue. Since the tongue is highly vascularized and contains large veins, especially in its inferior surface, bleeding was a considerable risk. Consequently, these veins should be observed and avoided during the penetration of the tissue (Figure 3).

**Figure 4:**
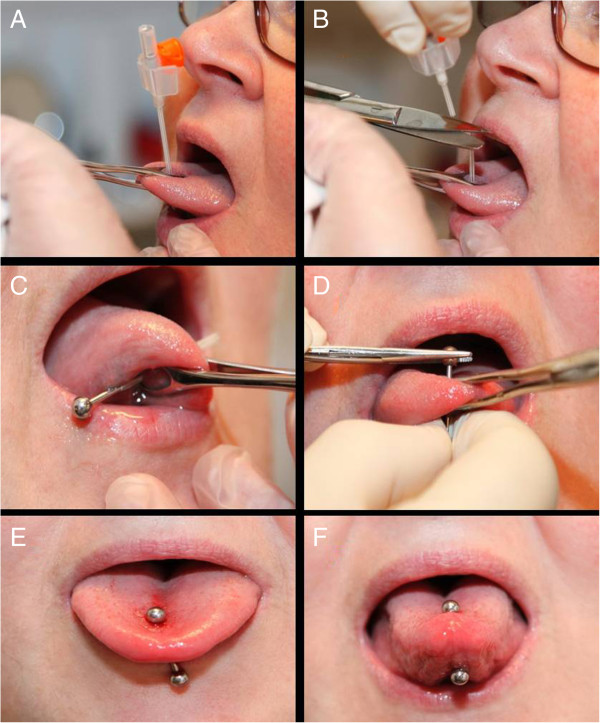
**The piercing procedure: (A) Penetration of the tongue from the upside by the Venflon needle; (B) After removal of the needle, the plastic tube remains in situ while the valve section is cut off and discharged; (C) The metal rod is guided through the tissue by means of the plastic tube; (D) The balls are tigthened onto the rod of the piercing by a needle holder while holding the tongue with the forceps; (E) Relaxed tongue muscles immidiately after the insertion, where the piercing is loosely attachted to the tongue; and (F) Contraction of the tongue muscles causes the piercing to become more firmly embedded (written consent for publication of these photos was obtained from the participant).**

At the insertion of the piercing, the tip of the tongue was held firmly with the tongue holder and a piece of cloth. Thus, a midline translingual canal could be prepared by penetrating the tongue with the canula of the venflon (Figure 4A). The canula was surrounded by a thin plastic tube. After the withdrawal of the canula and cutting off the valve section, a 30–40 mm piece of this plastic tube was left inside the tongue tissue (Figure 4B). This plastic tube subsequently served as a guide canal to insert the piercing rod, and the rod with the ball attached at one end was easily introduced through the plastic tube (Figure 4C). Finally, the plastic tube was removed and the second ball was screwed onto the rod and tightened by the needle holder (Figure 4D).

It was expected that the tongue would swell during the first days due to a reactive edema related to the tissue trauma of the procedure. Therefore, a longer piercing rod (approx. 20 mm) was used initially and during the healing period allowing for both swelling and free movement of the tongue muscles (Figure 4E,F). Finally, the subjects were instructed to rinse the tongue by means of a 0.1% chlorhexidine solution three times a day during the first week and an analgesic consisting of a 400 mg Ibuprofen tablet was prescribed 3 times during the first 24 hours.

### Evaluation of discomfort

The discomfort perceived by the participants was evaluated by a 100 mm Visual Analog Scale (VAS) having a linear score between 0 for no pain and 10 for the most intense pain imaginable. The participants ticked on the horizontal line equal to their perception [24-26]. The primary concern of the participants was pain, and thus, before the actual surgical procedure the participants were given a questionnaire regarding their expected perception of pain. In addition, the rating was performed immediately after the surgery and again two hours later. No data were collected regarding the perception of discomfort from local anesthetics since all participants declined this option.

Further evaluation of pain was recorded during the following ten days including additional factors of discomfort such as changes in sense of taste and speech, as well as the “degree of tangling with the teeth”. The participants scaled these modalities of discomfort once daily. Moreover, they were encouraged to note any additional complaints as well as other relevant events during the course of the piercing.

## Results

All participants declined the option of local anesthesia of the tongue since the discomfort of bilateral injections was considered larger than of the piercing itself. The insertion of the piercing was successfully performed in all four subjects as the procedure was simple and fast, and in each case completed in less than five minutes. The venflon system provided an excellent tool to both form the tissue canal and supplying a guiding tube for the titanium rod. No cases of bleeding or other acute complications were encountered. Swelling of the tongue was found in one subject only on the day after surgery where the tongue generally gained 5 mm in thickness for about 24 hours. The swelling was so moderate that the speech was unaffected and there was no embedding of the balls. Moreover, no cases of infection or injuries to the gingival mucosa were found.

During surgery no problems were encountered as to assemble the piercing rod and balls. However, during the healing period there was a tendency towards loosening of the balls, and in three cases a ball was lost at various points during the healing period. In one case the ball dropped out of the mouth several weeks later. This particular ball is thought to have been lodged in the piriform sinus.

**Figure 5:**
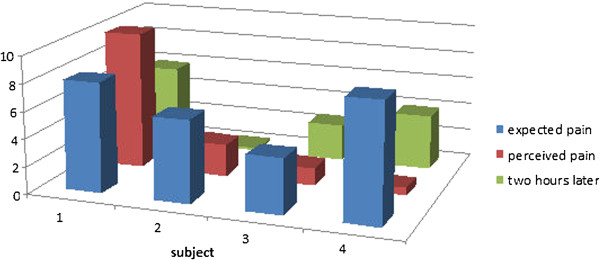
Expected pain, perceived pain and post-surgical pain two hours after surgery (10 cm VAS scale).

The pain experienced during the piercing procedure was in three cases lower and in one case higher than the expected pain. In all cases, the pain was lower after 2 hours than expected before the piercing procedure (Figure 5).

**Figure 6:**
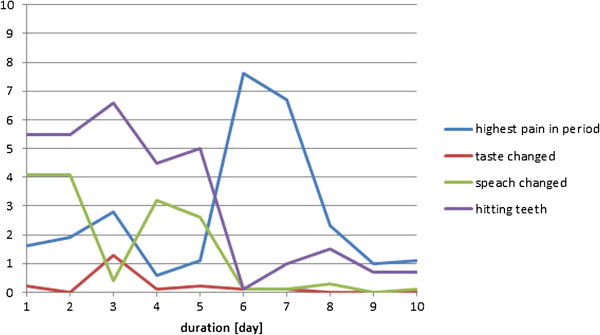
**Subject 1 (♂, age 59, tetraplegia after cervical fracture): Perception of pain, taste, speech, and problems with hitting the teeth with the piercing seen over a 10-day period after the first day of surgery (day 0).** At day 6 the piercing was changed (10 cm VAS scale).

**Figure 7:**
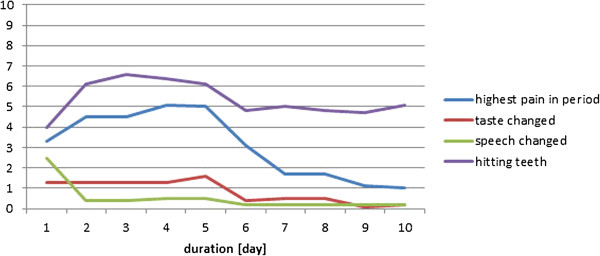
Subject 2 (♀, age 45; tetraplegia after cervical fracture): Perception of pain, taste, speech, and problems with hitting the teeth with the piercing seen over a 10-day period after the first day of surgery (day 0) (10 cm VAS scale).

**Figure 8:**
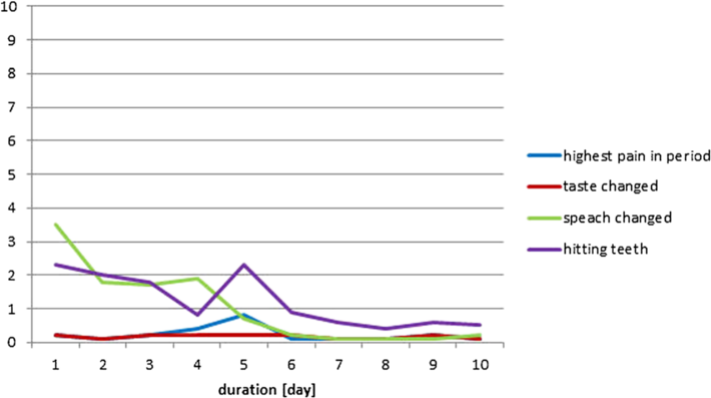
Subject 3 (♀, age 49; tetraplegia after severe early childhood meningitis): Perception of pain, taste, speech, and problems with hitting the teeth with the piercing seen over a 10-day period after the first day of surgery (day 0) (10 cm VAS scale).

**Figure 9:**
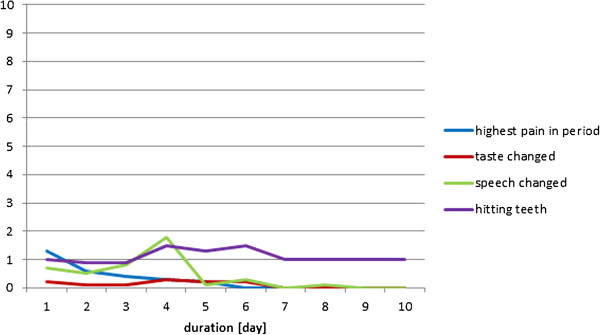
Subject 4 (♀, age 59; tetraplegia due to benign medullar glioma in C1 to C2 level): Perception of pain, taste, speech, and problems with hitting the teeth with the piercing seen over a 10-day period after the first day of surgery (day 0) (10 cm VAS scale).

The individual results from the four participants during the healing period have been displayed in Figures 6, 7, 8, and 9. In general, the results showed that after the 5^th^ day the problems with pain, sense of taste and speech had almost disappeared. Further, the problems with hitting the teeth with the balls had also diminished. However, in one case with higher complaints of hitting the teeth, the piercing had to be changed at day 5, which resulted in increased pain level during the next two days (Figure 6); in another case the same complaints remained at a relatively high level during the 10 day period (Figure 9). There were no additional complaints reported by the participants during the course of the piercing.

## Discussion

A medical tongue piercing technique has not previously been described or evaluated in the literature. This paper describes a new technique which is fast, safe and tolerable to the participants. Thus, the discomfort perceived during the procedure was of milder nature and comparable with pain from injections, and very few minor complications were recorded which is in contrast to the literature where oral piercings are often connected with discomfort and complications [20,26-34].

### The surgical procedure and the surgical kit

The tools of the surgical kit including their modifications proved efficient for the purpose. Further, the usage of the venflon system served as an excellent utensil for the insertion of the piercing rod. Altogether this provided a rapid procedure which was completed in a few minutes. Thus, limited mechanical manipulation of the tongue was needed which is likely to have reduced any post-procedural formation of reactive swelling or edema.

### Determination of rod size and its placement

In all 4 subjects a rod length of 20 mm was eventually chosen which gave a leeway allowing some post-procedural swelling. At the insertion of the piercing rod, 8–10 mm of extra length compared to the thickness of the tongue was chosen. This resulted in a rod length of 20 mm allowing free movements of the tongue despite any post-procedural swelling. Similarly, a total length of 20 mm has been suggested by Vieira et al. [20]. This length in combination with 6 mm balls may also explain why no problems were encountered with embedded balls which have been reported in the literature [32]. However, in two cases replacement with shorter rods was needed: One case already on day five where significant tangling with the teeth was encountered (Figure 6), and in the other case with minor tangling, replacement was performed at the end of the healing period (Figure 9). In both cases the tangling problems disappeared reducing the risks of any injuries to the teeth [35].

The piercings were all inserted in the midline approximately 20 mm from the tip of the tongue. This compromise between the ability to perform a good control of the system without drifting of the rod turned out to be a successful choice. In all cases the subjects had sufficient control of the anterior contacts of the palate dental brace [12,15], and further, no cases of drifting were encountered.

### Local anesthesia, pain, and analgesics

**Figure 10:**
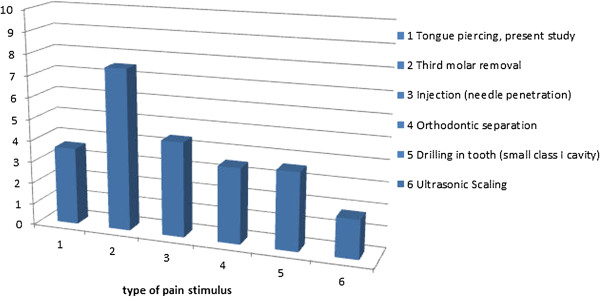
The mean perceived pain from tongue piercings in the current study compared with various other known oral pain stimuli.

No results regarding the use of local anesthesia were available and, therefore, the question of its effects as well as the procedural pain perceived during the insertion of the piercing was not addressed. It may be relevant that participants are given the option of an injection of local anesthesia because pain and anxiety control has previously been argued for in piercing situations where the participants have, for instance, heart conditions [20]. However, the mean procedural pain in this study was comparable with injections [27], ultrasonic cleaning of teeth [28], orthodontic separation of teeth [29], and preparation for small dental cavities [30], whereas it is far below the pain perceived after removal of a third molar [31]. For comparison, these situations are illustrated in Figure 10. Thus, based on these findings it can be documented that the piercing procedure described here caused pain in line with standard orthodontic procedures and injections. Therefore, it may be recommended to future participants that local anesthesia is not needed because its usage will not counterbalance its advantages. Moreover, future participants can be informed that any pain or discomforts will disappear more or less in four to six days, which has also been reported by another study [32]. The analgesics prescribed included only the first 24-hour period, and no additional prescriptions were requested. Thus the pain had significantly decreased and become tolerable during this period.

### Early complications and biocompatibility

Early complications were basically not seen in this series of piercings except a minor swelling in one subject. Bleeding was avoided by identifying the larger veins on the inferior side of the tongue; in oral piercings bleeding is a common problem and varies between 9 and 69% of the cases [20,21,32]. Moreover, no cases of infection, formation of reactive tissue, and allergic reaction were observed which can be explained by an almost non-traumatic and fast procedure applied with sterile instruments and piercing components as well as use of biocompatible material for these components. Finally, chlorhexidine was used during the healing period to maintain a clean environment in the mouth.

Overall complications in tongue piercings have been reported to be 53% in a group of British young people aged 16–24 years with the piercings being undertaken by non-medical personnel. In this study 26% subsequently had to seek professional help and 1% was hospitalized. Lack of hygienic measures may probably explain the majority of these cases [35], but also the lack of anatomical knowledge may contribute [20].

In this study the subjects were kept for observation for 24 hours at the hospital, thus offering professional assistance in cases of serious bleeding or swelling. However, the courses of the subjects were essentially uneventful. Thus, it can be concluded that future insertions of medical piercings may be performed in an out-patient setting.

After the overall study period a second oral examination was performed where no enamel cracks, fractures or gingival recessions were observed. Such late complications are recorded in significant numbers of cases where piercings have been used for a period of around four years [22]. Thus, the participants in this study may also have been exposed to such risks if the use of the piercings had been continued. Everyday use is expected for this product and will require regular adjustment of the piercing length to avoid hitting of the teeth, since too long piercing rods may result in dental cracks and gingival recessions [19]. Regular oral dental examinations should be recommended in order to monitor dental or mucosal injuries which must be expected as minor injuries to enamel and gingiva which have been reported in up to 80% of the cases [33].

The oral piercing needs to be cleaned on a regular basis [22] depending on an individual demand, and the participants would rely on the help of their personal assistants as for any other personal hygiene. This cleaning may be performed by an ordinary toothbrush without removal of the piercing. However, the piercing may easily be removed for further cleaning by the assistants, if needed. Altogether, cleaning of the piercing could be considered a minor problem.

### Overall safety and long-term complications

Safety has been a major concern for this group of susceptible subjects, and the participants were encouraged to note any events or complications during the study period. However, in addition to the early complications discussed above, no significant long-term complications were recorded. Especially, no cases of aspiration or swallowing of the balls were encountered. In the event of aspiration or swallowing of parts of the piercing, these parts could easily be detected by a conventional radiological examination. Whereas the swallowing of piercing parts should not result in further precautions, the aspiration should be followed by endoscopic examination of the lungs and removal of the foreign body to prevent later complications such as pneumonia and atelectasis of the lungs. Serious obstruction of the airways was not possible due to the small sizes of the piercing parts.

During the period of the study, all subjects reported loss of their balls at various times. In three cases the balls were recovered again more or less immediately, whereas in one case a ball was lost and not recovered immediately. In this case the ball was recovered several weeks later from the subject’s throat related to a coughing episode while the subject was lying face down in her bed. The only obvious explanation seemed to be that the ball had been maintained during this period at the piriform sinus. This may be possible if the dimensions of the ball fit exactly to this pharyngeal pouch and because this area has a lower sensitivity than the major part of the throat. The episode must be considered quite accidental although it poses a potential risk of aspiration. Consequently, dental glue (LOCTITE® M-121HP™) was introduced at the mounting of the balls in order to secure them further from loosening. After this additional measure no balls were lost. Moreover, the participants were provided with packages of extra balls which could be mounted by their personal assistants or family members.

## Conclusions

The study was limited by a smaller sample of participants which was explained by limitations in the recruitment. The condition of tetraplegia itself is seldom, and, moreover, normal tongue function as well as normal cognitive skills were demanded. Further, these subjects may have impaired motivation due to restricted personal resources, and participation in the overall study including a three month testing program may have seemed too demanding.

The insertion procedure of the medical tongue piercings in the tetraplegic subjects was simple, fast and tolerable. This resulted in minor trauma of the tongue tissue developing only minor edema in one of four subjects. Thus, the procedure can be considered safe. This means that the procedure may be performed on an out-patient basis without 24-hours hospital observation. The pain at the insertion of the piercing was mild and comparable with pain in connection with an ordinary injection. No significant early or long-term complications were encountered including dental or mucosal injuries during the three month period. The loosening of balls can be prevented by the application of dental glue. However, extra balls should be provided for replacements.

## Competing interests

The authors declare that they do not have competing interests.

## Authors’ contributions

BB participated in the planning of the protocol, the development of the surgical kit, the selection and information of participants, the clinical procedure, the course of observations, the evaluation of discomforts and the practical and technical procedures related to the piercings. MG participated in the planning of the protocol for the selection of participants, the clinical procedure, the course of observations, and the evaluation of discomforts as well as the practical procedures related to the piercings. ERL participated in the development of the usage of a piercing in a tongue control system. LNSAS raised funding for the study, participated in the planning of the protocol, the development of the usage of a piercing in a tongue control system, the selection of participants and in the planning of the clinical procedure and in the course of observations related to the direct contact to the subjects. All authors read and approved the final manuscript.

## References

[B1] Lau C O'Leary S Comparison of computer interface devices for persons with severe physical disabilities Am J Occup Ther 1993 11 1022 1030 8279497 10.5014/ajot.47.11.1022

[B2] Simpson T Gauthier M Prochazka A Evaluation of tooth-click triggering and speech recognition in assistive technology for computer access Neurorehab Neural Repair 2010 2 188 194 10.1177/1545968309341647 19679651

[B3] Koester HH User performance with speech recognition: a literature review Assist Technol 2001 13 116 130 10.1080/10400435.2001.10132042 12530839

[B4] Hansen JP Tørning K Johansen AS Itoh K Aoki H Gaze typing compared with input by head and hand Proceedings of the Eye Tracking Res Appl Sym 2004, ETRA 2004. March 22-24, San Antonio Texas USA 2004 New York: ACM Press 131 138

[B5] Hansen DW Ji Q In the eye of the beholder: a survey of models for eyes and gaze IEEE Trans Pattern Anal Mach Intell 2010 3 478 500 20075473 10.1109/TPAMI.2009.30

[B6] Cincotti F Mattia D Aloise F Bufalari S Schalk G Oriolo G Cherubini A Marciani MG Babiloni F Non-invasive brain-computer interface system: Towards its application as assistive technology Brain Res Bull 2008 6 796 803 18394526 10.1016/j.brainresbull.2008.01.007 PMC2896271

[B7] Ghovanloo M Tongue Operated Assistive Technologies Proceedings of the 29th Annual International Conference of the IEEE EMBS Cité Internationale, August 23-26 2007 France: Lyon 4376 4379 10.1109/IEMBS.2007.4353307 18002973

[B8] Kim D Tyler ME Beebe DJ Development of a tongue-operated switch array as an alternative input device Int J Hum-Comput Int 2005 18 19 38 10.1207/s15327590ijhc1801_2

[B9] Christensen CS Oral controller method and apparatus US Patent 1991 5 233 662

[B10] Saponas TS Kelly D Babak A Parviz BA Tan DS Optically Sensing Tongue Gestures for Computer Input Proceedings of the User Interface Soft Technol Sym 2009, UIST’09. October 4–7, Victoria BC, Canada 2009 New York: ACM Press 177 180

[B11] Huo X Wang J Ghovanloo M A magneto-inductive sensor based wireless tongue-computer interface IEEE Trans Neural Sys Rehab Eng 2008 5 497 504 10.1109/TNSRE.2008.2003375 PMC4470907 18990653

[B12] Andreasen Struijk LNS An inductive tongue computer interface for control of computers and assistive devices IEEE Trans Biomed Eng 2006 53 2594 2597 17152438 10.1109/TBME.2006.880871

[B13] Lontis ER Struijk LN Design of inductive sensors for tongue control system for computers and assistive devices Disabil Rehabil Assist Technol 2010 5 266 271 10.3109/17483101003718138 20307253

[B14] Caltenco HA Lontis ER Bentsen B Andreasen Struijk LNS Effects of sensory feedback in intra-oral target selection tasks with the tongue Disabil Rehabil Assist Technol 2013 4 330 339 22779705 10.3109/17483107.2012.699991

[B15] Lontis ER Lund ME Christensen HV Bentsen B Gaihede M Caltenco HA Andreasen Struijk LNS Clinical evaluation of wireless inductive tongue computer interface for control of computers and assistive devices Proceedings of the 32nd Annual International Conference of the IEEE Engineering in Medicine and Biology Society: 31 August – 4 September 2010 2010 Buenos Aires: IEEE Press 3365 3368 10.1109/IEMBS.2010.5627924 21097236

[B16] Boudreau S Lontis R Caltenco Arciniega HA Svensson P Sessle BJ Andreasen Struijk LNS Arendt-Nielsen L Features of cortical neuroplasticity associated with multidirectional novel motor skill training: a TMS mapping study Exp Brain Res 2013 4 513 526 23307156 10.1007/s00221-012-3391-2

[B17] Caltenco HA Lontis ER Boudreau SA Bentsen B Struijk J Andreasen Struijk LNS Tip of the tongue selectivity and motor learning in the palatal area IEEE Trans Biomed Eng 2012 59 174 182 21954196 10.1109/TBME.2011.2169672

[B18] Lontis R Caltenco HA Bentsen B Christensen HV Lund ME Andreasen Struijk LNS Inductive pointing device for tongue control system for computers and assistive devices Proceedings of the 31st Annual International Conference of the IEEE Engineering in Medicine and Biology Society, 2–6 September 2009 2009 Minneapolis, USA: IEEE 2380 2383 10.1109/IEMBS.2009.5334985 19965193

[B19] Lontis ER Struijk LN Alternative design of inductive pointing device for oral interface for computers and wheelchairs Proceedings of the 32nd Annual International Conference of the IEEE Engineering in Medicine and Biology Society: 28 August – 1 September 2012 2012 San Diego: IEEE Press 3328 3331 10.1109/EMBC.2012.6346677 23366638

[B20] Vieira EP Ribeiro AL Pinheiro J de JV Alves S de M Jr Oral piercings: immediate and late complications J Oral Maxillofac Surg 2011 69 3032 3037 10.1016/j.joms.2010.12.046 21550157

[B21] Bone A Ncube F Nichols T Noah ND Body piercing in England: a survey of piercing at sites other than earlobe BMJ 2008 336 1426 1428 10.1136/bmj.39580.497176.25 18556275 PMC2432173

[B22] Ziebolz D Hildebrand A Proff P Rinke S Hornecker E Mausberg RF Long-term effects of tongue piercing–a case control study Clin Oral Investig 2012 16 231 237 10.1007/s00784-011-0510-6 21271349 PMC3259306

[B23] Yamazoe J Nakagawa M Matono Y Takeuchi A Ishikawa K The development of Ti-alloys for dental implant with high corrosion resistance and mechanical strength Dent Mater J 2007 26 260 267 10.4012/dmj.26.260 17621943

[B24] Wewers ME Lowe NK A critical review of visual analogue scales in the measurement of clinical phenomena Res Nurs Health 1990 13 227 236 10.1002/nur.4770130405 2197679

[B25] Chapman CR Casey KL Dubner R Foley KM Gracely RH Reading AE Pain measurement: an overview Pain 1985 22 1 31 4011282 10.1016/0304-3959(85)90145-9

[B26] Chéry-Croze S Relationship between noxious cold stimuli and the magnitude of pain sensation in man Pain 1983 15 265 269 10.1016/0304-3959(83)90061-1 6856323

[B27] Bergius M Berggren U Kiliaridis S Experience of pain during an orthodontic procedure Eur J Oral Sci 2002 110 92 98 10.1034/j.1600-0722.2002.11193.x 12013568

[B28] Svensson P Petersen JK Svensson H Efficacy of a topical anesthetic on pain and unpleasantness during scaling of gingival pockets Anesth Prog 1994 41 35 39 8638858 PMC2148810

[B29] Leavitt AH King GJ Ramsay DS Jackson DL A longitudinal evaluation of pulpal pain during orthodontic tooth movement Orthod Craniofac Res 2002 5 29 37 10.1034/j.1600-0544.2002.01158.x 12071371

[B30] Bentsen B Wenzel A Svensson P Comparison of the effect of video glasses and nitrous oxide analgesia on the perceived intensity of pain and unpleasantness evoked by dental scaling Eur J Pain 2003 7 49 53 10.1016/S1090-3801(02)00051-4 12527317

[B31] Nørholt SE Treatment of acute pain following removal of mandibular third molars. Use of the dental pain model in pharmacological research and development of a comparable animal model Int J Oral Maxillofac Surg 1998 27 1 41 9638499 10.1016/s0901-5027(98)80001-5

[B32] López-Jornet P Navarro-Guardiola C Camacho-Alonso F Vicente-Ortega V Yánez-Gascon J Oral and facial piercings: a case series and review of the literature Int J Dermatol 2006 45 805 809 10.1111/j.1365-4632.2006.02743.x 16863515

[B33] Garcia-Pola MJ Garcia-Martin JM Varela-Centelles P Bilbao-Alonso A Cerero-Lapiedra R Seoane J Oral and facial piercing: associated complications and clinical repercussion Quintessence Int 2008 39 51 59 18551217

[B34] De Moor R De Witte A Delmé K De Bruyne M Hommez G Complications dentaires et buccales des piercing labiaux et lingaux Rev Belge Med Dent 2007 62 104 112 18506959

[B35] Campbell A Moore A Williams E Stephens J Tatakis DN Tongue piercing: impact of time and barbell stem length on lingual gingival recession and tooth chipping J Periodontol 2002 73 289 297 10.1902/jop.2002.73.3.289 11922258

